# Mechanism-informed diagnostic accuracy of blood biomarkers for sepsis-associated encephalopathy: a systematic review and Bayesian diagnostic network meta-analysis

**DOI:** 10.3389/fnmol.2026.1789013

**Published:** 2026-08-03

**Authors:** Qian Zhang, Runying Zhu, Yi Li, Hui Li, Lixia Liu, Zhenjie Hu, Yan Huo

**Affiliations:** 1 Department of Intensive Care Unit, The Fourth Hospital of Hebei Medical University, Shijiazhuang, China; 2 Department of Anesthesiology, Hebei General Hospital, Shijiazhuang, China

**Keywords:** Bayesian network meta-analysis, biomarkers, blood–brain barrier, delirium, diagnostic accuracy, glial markers, immune activation, sepsis-associated encephalopathy

## Abstract

**Background:**

Sepsis-associated encephalopathy (SAE) is a common and devastating manifestation of acute brain dysfunction in sepsis, yet mechanism-informed blood biomarkers with clinically interpretable diagnostic accuracy remain uncertain. A growing range of candidates spanning innate immune activation, blood–brain barrier dysfunction, glial response, and neuronal injury has been reported, but their comparative diagnostic performance and biological hierarchy are unclear, partly due to heterogeneity in phenotyping and sampling timing.

**Main body:**

We performed a PRISMA/PRISMA-DTA-compliant systematic review and comparative diagnostic evidence synthesis, including a Bayesian diagnostic network meta-analysis, to prioritize blood-based biomarkers for SAE. PubMed, Web of Science, EMBASE, Cochrane Library, CNKI, VIP, and WFSD were searched from inception to October 2025. Studies were eligible if SAE case definitions and extractable (or reconstructable) 2 × 2 diagnostic data were available. To characterize heterogeneity, SAE reference standards were tiered (delirium-focused tools vs. broader encephalopathy definitions vs. unclear/mixed), and biomarker sampling was captured using an anchor-based context (anchor event and time-from-anchor). Comparative ranking in the Bayesian diagnostic network meta-analysis was based on the Advantage Index (S-value) derived from posterior distributions. Sixteen studies (*n* = 1,666) were included, covering biomarkers along an immune–BBB/glia–neuron axis. In pooled diagnostic test accuracy synthesis, blood–brain barrier/glial markers (e.g., GFAP, S100β) generally showed more balanced discrimination than classic neuronal injury markers in the included diagnostic settings. In Bayesian comparative ranking, upstream immune/alarmin candidates (TRAF6, S100A8) and day-3 S100β ranked highest; however, several top-ranked nodes were supported by sparse evidence (single-study nodes), and thresholds/platforms and sampling contexts were study-specific. Importantly, few included studies evaluated whether biomarkers add diagnostic information beyond established clinical predictors (e.g., illness severity scores and sedation- or metabolic-related confounders), limiting immediate clinical applicability.

**Short conclusion:**

Blood biomarkers for SAE exhibit a biologically coherent immune-to-neuronal cascade, and their diagnostic performance appears sensitive to phenotyping and anchor-based sampling context. These findings are hypothesis-generating and support prospective head-to-head validation to determine whether mechanism-staged biomarker panels add incremental diagnostic value beyond established clinical predictors under standardized phenotyping and anchor-based sampling protocols.

**Systematic review registration:**

https://www.crd.york.ac.uk/PROSPERO/view/CRD42024531398, CRD42024531398.

## Background

Sepsis-associated encephalopathy (SAE) is a frequent manifestation of acute brain dysfunction in sepsis and remains clinically challenging because it lacks focal neurological signs and often overlaps with sedation- and metabolic-related mental status changes (Mazeraud et al., 2020). Rather than being solely a downstream consequence of neuronal injury, mounting evidence supports SAE as a neuroimmune disorder driven by systemic inflammation and peripheral-to-central immune signaling (Binuya et al., 2022; Gao and Hernandes, 2021). Innate immune activation and alarmin release promote endothelial dysfunction, microcirculatory impairment, and blood–brain barrier (BBB) disruption, which in turn amplifies neuroinflammation through microglial activation and astrocytic reactivity (Kuperberg and Wadgaonkar, 2017; Barichello et al., 2021). These events can culminate in synaptic failure, neurotransmission imbalance, and diffuse encephalopathy, ultimately contributing to increased short- and long-term mortality, prolonged ICU stay, and persistent cognitive impairment (Hong et al., 2023). In this context, candidate biomarkers should ultimately be evaluated not only for standalone discrimination but also for whether they provide incremental diagnostic value beyond established clinical predictors and routine bedside assessments.

Damage-associated molecular patterns (DAMPs)/alarmins, including S100A8 (calprotectin) (Wang et al., 2018), may capture upstream inflammatory signaling relevant to SAE. S100A8 can engage TLR4-related pathways involving TRAF6 and downstream cytokine responses (e.g., IL-1β, IL-6, TNF-α) (Zhang et al., 2016; Dolmatova et al., 2021), which have been linked to endothelial activation and BBB vulnerability. Accordingly, upstream immune mediators may provide an earlier window into SAE-related pathobiology than markers reflecting established neuroglial or neuronal injury.

A broad range of blood-based biomarkers has been investigated in SAE, spanning inflammatory mediators and innate immune signaling molecules (upstream), BBB/glial injury markers (intermediate), and neuronal/axonal injury markers (downstream) (Scarlatescu et al., 2025). However, the evidence remains fragmented. Many studies report between-group differences without extractable 2 × 2 diagnostic data or standardized thresholds, limiting bedside interpretability. In addition, SAE phenotyping and reference standards vary across studies, and emerging innate immune/alarmin candidates are rarely evaluated within a unified, head-to-head comparative diagnostic framework (Zenaide and Gusmao-Flores, 2013). As a result, clinicians and researchers still lack an evidence-informed prioritization of biomarkers aligned with the neuroimmune pathogenesis and anchor-based sampling context of SAE.

**Figure 1:**
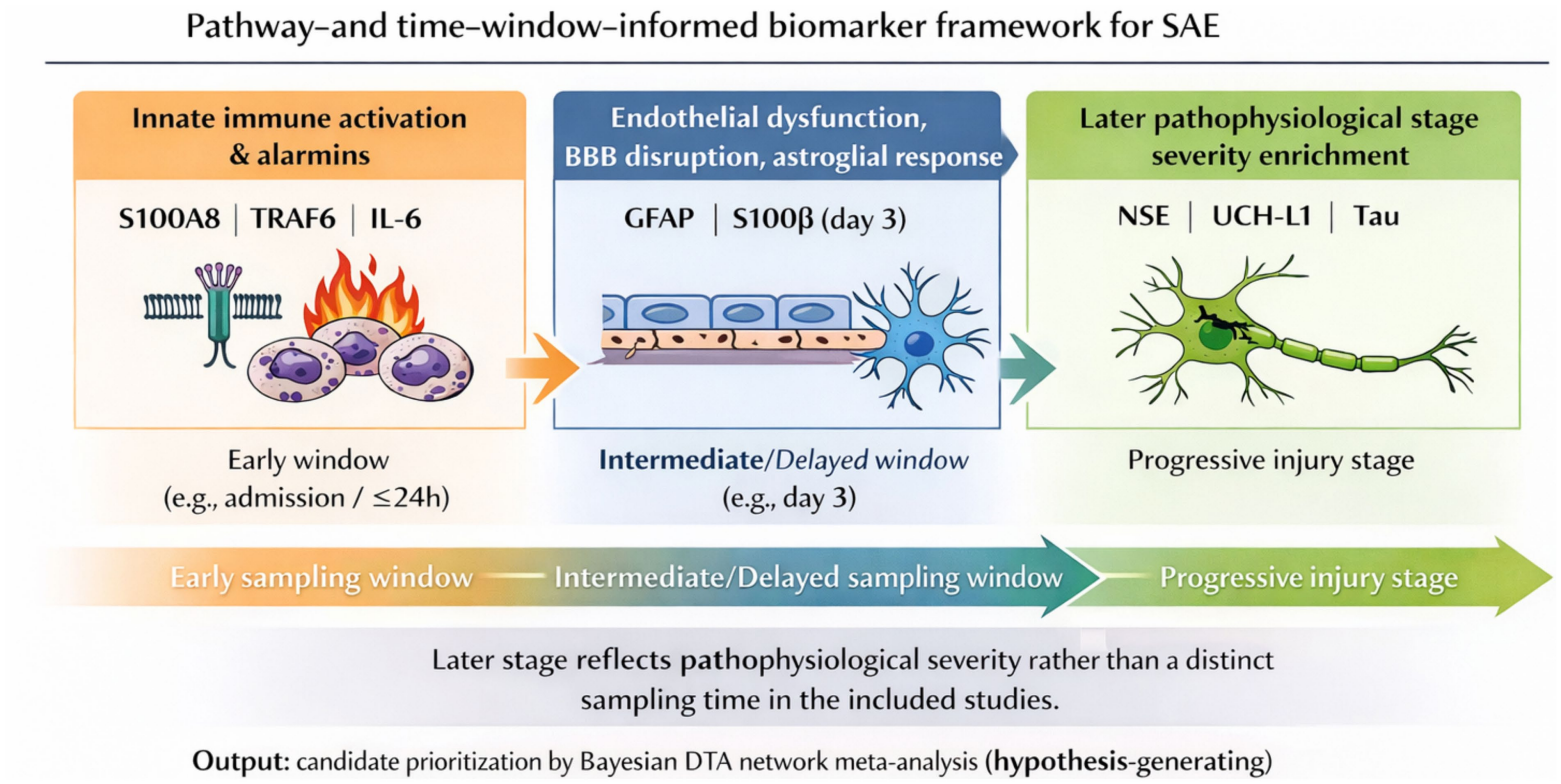
Pathway- and stage-informed biomarker framework for sepsis-associated encephalopathy (SAE). Candidate biomarkers are organized along the evolving pathophysiological cascade of SAE, from innate immune activation and alarmin release (e.g., S100A8, TRAF6, IL-6), through endothelial dysfunction, blood–brain barrier disruption, and astroglial response (e.g., GFAP, S100β), to downstream neuronal/axonal injury and synaptic dysfunction (e.g., NSE, UCH-L1, Tau). Although most included studies measured blood-based biomarkers early after clinical anchor events (typically within 72 h), the stratification shown here reflects biological hierarchy rather than absolute sampling time, with neuronal injury markers representing downstream disease severity rather than later blood sampling. This framework informed hypothesis generation and candidate prioritization in the subsequent Bayesian diagnostic network meta-analysis.

Accordingly, we performed a PRISMA/PRISMA-DTA–compliant systematic review of diagnostic test accuracy studies and a Bayesian diagnostic network meta-analysis to compare and prioritize blood-based biomarkers for SAE within an immune–BBB/glia–neuron axis. We extracted or reconstructed study-level 2 × 2 data and captured sampling anchor events and time-from-anchor windows to contextualize diagnostic performance. Given heterogeneity in SAE phenotyping and index-test thresholds, and sparse evidence for several candidates, comparative rankings are presented as hypothesis-generating. Future prospective head-to-head cohorts should determine whether candidate biomarkers provide incremental diagnostic value beyond established clinical predictors (e.g., delirium tools, severity scores, and sedation/exclusion workflows) under standardized phenotyping and anchor-based sampling protocols (Figure 1).

## Methods

We conducted this systematic review and diagnostic evidence synthesis in accordance with the Preferred Reporting Items for Systematic Reviews and Meta-Analyses (PRISMA) statement (Page et al., 2021) and PRISMA-DTA guidance (McInnes et al., 2018). We prespecified an interpretive framework that positions blood-based biomarkers along a mechanistic immune–BBB/glia–neuron axis to support clinically meaningful prioritization. Given heterogeneity in SAE phenotyping, biomarker platforms, and sampling practices, our primary objective was comparative prioritization (hypothesis-generating) rather than immediate clinical implementation. A completed PRISMA/PRISMA-DTA checklist is provided in Supplementary material.

### Protocol and registration

The study protocol was registered in PROSPERO (CRD42024531398). The review question followed a diagnostic test accuracy (DTA) framework (Bossuyt et al., 2023): adult sepsis populations; sepsis-associated encephalopathy (SAE) versus septic controls without encephalopathy, including studies using alternative labels such as sepsis-associated acute brain dysfunction; blood-based index tests covering inflammatory/innate immune candidates and neuroglial/neuroaxonal injury markers; and study-level diagnostic accuracy outcomes.

#### Eligibility criteria

Participants. Adults (≥18 years) with sepsis, severe sepsis, or septic shock (as defined by each study) (Singer et al., 2016).

Target condition. Sepsis-associated encephalopathy (SAE), defined by each study. Studies using alternative labels, such as sepsis-associated acute brain dysfunction, were mapped to the SAE framework for eligibility assessment and reference-standard tiering. As no universally accepted gold standard exists, we prespecified a reference-standard tiering to transparently characterize heterogeneity (Chung et al., 2020):

- Tier 1 (delirium-focused phenotyping): delirium screening tools (e.g., CAM-ICU, ICDSC) used as the primary criterion (Ely et al., 2001; Bergeron et al., 2001);
- Tier 2 (broader encephalopathy phenotyping): encephalopathy defined with explicit exclusion of structural CNS disease and/or incorporation of neurological assessment beyond delirium screening (± EEG/imaging as reported) (Chung et al., 2020);
- Tier 3 (mixed/unclear): mixed criteria or insufficiently described phenotyping.

Reference-standard tiering was used as a transparency framework to characterize heterogeneity rather than to assume equivalence across phenotyping approaches. We retained all eligible tiers in the primary synthesis because restricting the analysis to a single phenotyping approach would have substantially reduced the already sparse evidence base and weakened network connectivity.

Index tests. Blood-based biomarkers measured in serum, plasma, or whole-blood–derived components (e.g., PBMC), including but not limited to TRAF6, S100A8, IL-6, GFAP, S100β, NSE, UCH-L1, and Tau. For biomarkers assessed at multiple timepoints (e.g., repeated S100β), each study–biomarker–timepoint combination was treated as a distinct node when extractable 2 × 2 data were available, with timing retained as part of the sampling-context definition.

Outcomes. Studies were eligible if diagnostic 2 × 2 data (TP/FP/FN/TN) were reported or could be reconstructed from sufficient information (e.g., sensitivity and specificity with group sizes and a stated cutoff).

Study designs. Prospective or retrospective observational studies. We excluded case reports, reviews, animal/*in vitro* studies, and studies without sufficient data for DTA reconstruction.

Language: English and Chinese.

### Information sources and search strategy

We searched PubMed, Web of Science, EMBASE, Cochrane Library, CNKI, VIP, and WFSD from inception to October 2025 using combinations of controlled vocabulary and free-text terms covering sepsis, sepsis-associated encephalopathy/septic encephalopathy, delirium/acute brain dysfunction, and candidate blood biomarkers (e.g., “GFAP,” “S100B,” “NSE,” “UCH-L1,” “Tau,” “S100A8,” “TRAF6,” “IL-6”). Reference lists of eligible studies and relevant reviews were also screened. Gray literature sources and conference abstracts were not systematically included because this review was designed as a diagnostic test accuracy synthesis requiring extractable or reconstructable 2 × 2 data and sufficiently reported thresholds and methods, which are rarely available in abstract-only reports. The complete database-specific search strategies are provided in Supplementary material.

### Study selection

Two reviewers independently extracted data using a prespecified standardized form: author, year, country, design, clinical setting, sample size (SAE/non-SAE), sepsis definition, SAE phenotyping criteria (reference-standard tier), specimen type (serum/plasma/whole-blood–derived), assay/platform (e.g., ELISA, chemiluminescence), and index-test threshold (cutoff) when available. Where reported, we additionally extracted study-level contextual variables that could influence biomarker interpretation, including clinical setting/severity context, sedation handling, and delirium assessment practices/frequency (Page et al., 2021).

### Data extraction and operational definitions

Two reviewers independently extracted data using a prespecified standardized form: author, year, country, design, clinical setting, sample size (SAE/non-SAE), sepsis definition, SAE phenotyping criteria (reference-standard tier), specimen type (serum/plasma/whole-blood–derived), assay/platform (e.g., ELISA, chemiluminescence), and index-test threshold (cutoff) when available.

Anchor-based sampling context. To avoid over-standardization across heterogeneous study protocols, sampling was recorded as a two-part descriptor:

- sampling anchor event (as reported: ICU admission, hospital admission, sepsis diagnosis, CAM-ICU assessment, or enrollment), and
- time from anchor (hours/days or study-defined labels such as “Day 1/Day 3”). When “Day 1/Day 3” were used, we retained the authors’ labels unless explicit mapping to ~24 h/~72 h was stated. Anchor events and time-from-anchor windows are summarized in Table 1.

**Table 1:** Characteristics of included studies and diagnostic sampling framework for sepsis-associated encephalopathy.

First author (year)	Country	Study design	Sample size	SAE reference standard	Biomarker	Anchor event	Time from anchor	Specimen	Cutoff (unit)
Wu et al. (2019)	China	Prospective	105	CAM-ICU + clinical	BBB/Glia: GFAP	ICU admission	Day1	Serum	0.5322 μg/L
					Neuronal: UCH-L1				7.72 ng/mL
Zhang et al. (2016)	China	Prospective	57	CAM-ICU + clinical	Immune: S100A8	ICU admission	Day1	Plasma	1.93 ng/mL
					Immune: TRAF6			PBMC	1.44 (relative level)
					BBB/Glia: S100β			Serum	0.28 μg/L
					Neuronal: NSE			Serum	0.79 ng/mL
Erikson et al. (2019)	Finland	Prospective	22	CAM-ICU + clinical	BBB/Glia: S100β	CAM-ICU assessment	same day	Serum	0.15 ug/L
					Immune: IL-6				138.3 pg/mL
					Neurona: NSE				12.5 ng/mL
Nguyen et al. (2006)	Belgium	Prospective	170	GCS-based brain dysfunction	BBB/Glia: S100β	ICU admission	0–48 h	Serum	NR (paper reports “normal upper limit 0.5 μg/L”)
					Neuronal: NSE				NR (paper reports “normal upper limit 12.5 μg/L”)
Wu et al. (2020)	China	Prospective cohort	104	CAM-ICU + clinical	BBB/Glia: S100β (Day 1)	ICU admission	Day 1	Serum	0.226 μg/L
					BBB/Glia: S100β (Day 3)		Day 3		0.144 μg/L
Yao et al. (2014)	China	Prospective observational	112	CAM-ICU + clinical	BBB/Glia: S100β	ICU admission	Day 1	Serum	0.131 μg/L
					Neuronal: NSE				24.15 ng/mL
Cao et al. (2023)	China	Observational	100	CAM-ICU + clinical	BBB/Glia: GFAP	Hospital admission	Day 1	Serum	1.23 μg/L
					Neuronal: NSE				25 ng/mL
					BBB/Glia: S100β				0.203 μg/L
Feng et al. (2017)	China	Retrospective observational	59	CAM-ICU + clinical	Neuronal: NSE	ICU admission	Day 1	Serum	14.36 μg/L
					BBB/Glia: S100β (Day 3)		Day 3		0.14 μg/L
					Immune: IL-6		Day 1		91.305 pg/mL
Zhang et al. (2024)	China	Prospective cohort	101	CAM-ICU + RASS (SAE)	Neurona: NSE	Enrollment	≤72 h	Plasma	NR
					BBB/Glia: S100β				NR
Yan et al. (2019)	China	Retrospective observational	152	CAM-ICU + clinical	[BBB/Glia]GFAP	ICU admission	Day 1	Serum	0.67 μg/L
					Neuronal: NSE				15.57 μg/L
					BBB/Glia: S100β				0.29 μg/L
Jiang (2021)	China	Retrospective Observational	64	CAM-ICU + clinical	BBB/Glia: S100β	Admission (hospitalor ICU; as reported)	Day 1	Serum	0.1405 μg/L
Li et al. (2022)	China	Retrospective observational	42	CAM-ICU + clinical	Immune: IL-6	Sepsis diagnosis	Day 1	Serum	157 pg/mL
					Neuronal: Tau				6.4 pg/mL
Liu and Dai (2024)	China	Retrospective Observational	224	CAM-ICU + clinical	Neuronal: Tau	Sepsis diagnosis	Day 1	Serum	9.78 pg/mL
Tan et al. (2024)	China	Retrospective Observational	177	CAM-ICU + clinical	Neuronal: UCH-L1	Hospital admission	Day 1	Serum	7. 35 ng/mL
Yuan and Huang (2020)	China	Prospective observational	68	CAM-ICU + clinical	Neuronal: NSE	ICU admission	Day 1	Serum	16.89 ng/mL
					Immune: IL-6			Serum	88.5 pg/mL
Zhao et al. (2019)	China	Retrospective observational	109	CAM-ICU + clinical	Neuronal: Tau	Hospital admission	Day 1	Serum	15 pg/mL

Anchor event indicates the reported time reference point for blood sampling (e.g., ICU admission, sepsis diagnosis, or CAM-ICU assessment). Time from anchor is recorded as reported; Day 1/Day 3 refer to study-defined ICU timepoints (approximately 24 h/72 h when stated by authors). Cutoffs and units were typographically standardized (e.g., ng/mL, μg/L, pg/mL) while preserving study-reported values. ^†^TRAF6 was quantified in PBMCs as a semi-quantitative relative expression (not a plasma/serum concentration).

Handling of thresholds. When multiple cutoffs were reported, we prioritized prespecified or clinically implemented thresholds (Cohen et al., 2016). If only receiver-operating characteristic (ROC)-derived thresholds were available, we extracted the authors’ primary reported threshold; if several ROC thresholds were presented without clear priority, we recorded the threshold associated with the highest Youden index and documented this decision for transparency.

Reconstruction of 2 × 2 data. For each study–biomarker pair, diagnostic contingency data (TP, FP, FN, TN) were extracted directly or reconstructed from reported sensitivity, specificity, and sample size information when necessary. When reconstruction was not uniquely identifiable from published information, the datapoint was not entered into quantitative synthesis and was documented in Supplementary Table S1. The complete 2 × 2 dataset and associated anchor-based timing information are provided in Supplementary Table S1.

### Risk of bias and applicability

Risk of bias and applicability concerns were assessed using QUADAS-2 across four domains (patient selection, index test, reference standard, and flow/timing) (Whiting et al., 2011). Two reviewers independently judged each domain; discrepancies were resolved by discussion. We considered flow/timing and index-test thresholding as key applicability issues given heterogeneous anchor-based sampling and assay platforms.

### Statistical analysis and evidence synthesis

We synthesized diagnostic accuracy using hierarchical DTA models. When at least two studies evaluated the same biomarker under comparable conditions, we summarized pooled sensitivity and specificity with corresponding uncertainty intervals (Reitsma et al., 2005; Rutter and Gatsonis, 2001).

Bayesian diagnostic network meta-analysis was performed within a hierarchical ANOVA framework using Markov chain Monte Carlo (MCMC) methods implemented in JAGS (version 4.3.2) via R (version 4.4.1; packages: rjags and gemtc). For each model, four chains were run with 50,000 iterations, including 20,000 burn-in iterations, and samples were thinned every 10 iterations. Weakly informative priors were applied for all basic parameters. Convergence was assessed using trace plots and the Gelman–Rubin statistic (R̂ < 1.05). Posterior distributions were summarized as medians with 95% credible intervals. Comparative ranking was based on the Advantage Index (S-value) derived from posterior distributions. Given heterogeneity in reference standards, thresholds, and sampling contexts, comparative estimates were interpreted as hypothesis-generating. Because the number of studies within individual biomarker nodes was limited and network connectivity was sparse, formal tier-specific network comparisons were not considered sufficiently robust. Accordingly, rankings were interpreted cautiously and used to inform prioritization rather than definitive clinical recommendations.

### Network assumptions and consistency

We examined network geometry and assessed the plausibility of transitivity by comparing study-level distributions of reference-standard tiers and anchor-based sampling windows across biomarkers. Formal inconsistency assessment (e.g., node-splitting) and deviance-based model comparisons were not reliably feasible because of sparse networks and limited study numbers for many nodes; this limitation was considered when interpreting comparative rankings.

Small-study effects/publication bias. Formal assessment of small-study effects (e.g., Deeks’ test) was not performed because of the limited number of studies per biomarker.

Sensitivity analyses. Because the number of studies per biomarker node was limited and phenotyping/sampling heterogeneity was substantial, formal sensitivity analyses restricted by reference-standard tier or sampling context were not considered sufficiently robust for stable comparative inference. We therefore used prespecified reference-standard tiering and anchor-based sampling descriptors to support cautious interpretation of pooled and network estimates. Biomarkers supported by single-study evidence (e.g., TRAF6, S100A8) were explicitly flagged as exploratory nodes and interpreted cautiously.

Reporting. Study characteristics, biomarker pathway grouping, and anchor-based sampling context are presented in Table 1. Additional study-level contextual information, including clinical setting/severity context, sedation handling, and delirium assessment/frequency where available, is summarized in Supplementary Table S2. Study-level reconstructed diagnostic contingency data (TP, FP, FN, TN) used for quantitative synthesis are provided in Supplementary Table S1.

## Results

### Study selection and study characteristics

**Figure 2:**
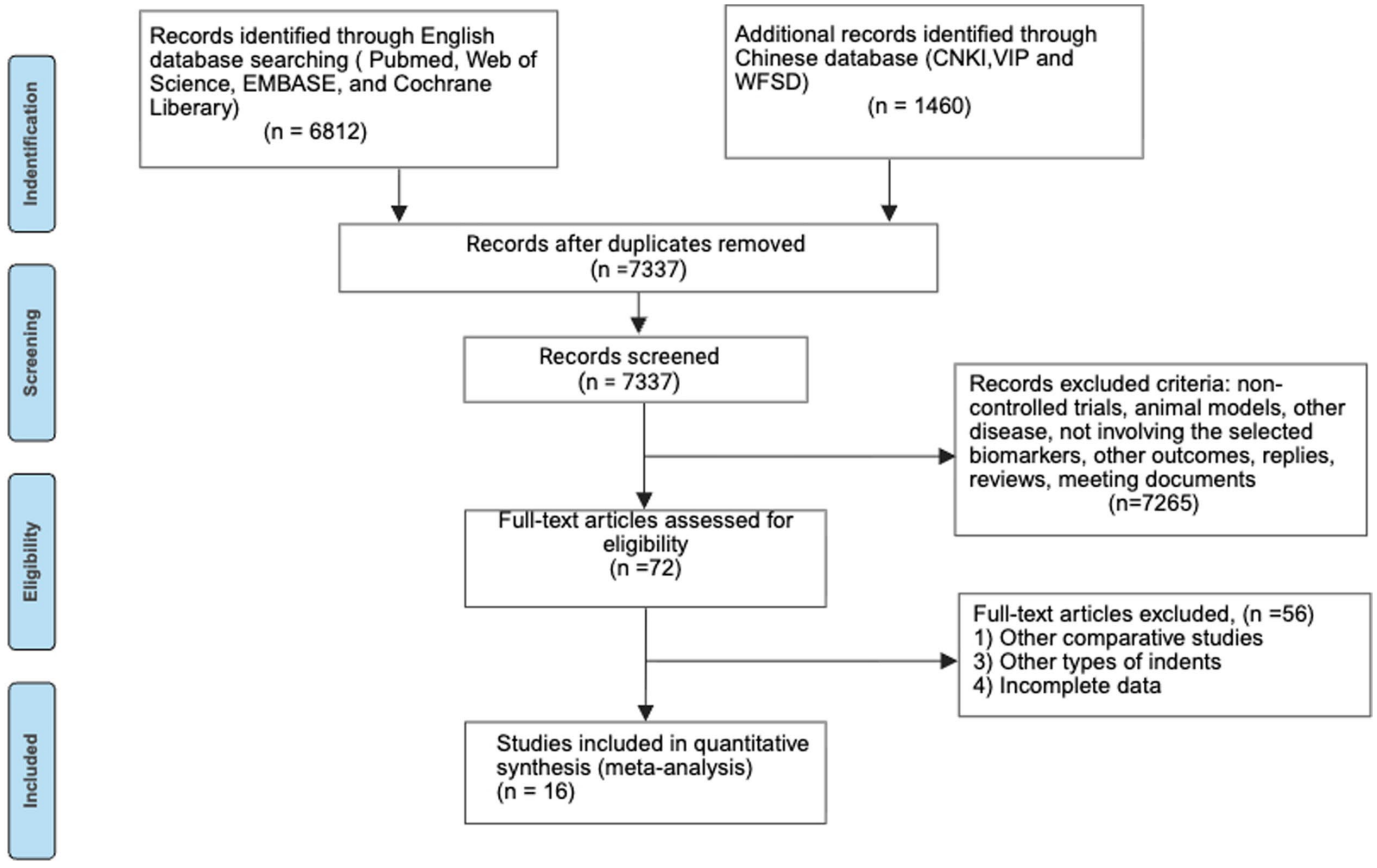
PRISMA flow diagram of literature selection process for the diagnostic network meta-analysis of SAE biomarkers.

The systematic search identified 8,272 records (6,812 from international databases and 1,460 from CNKI, VIP, and WFSD). After deduplication, 7,337 titles/abstracts were screened and 72 full texts were assessed. Sixteen studies met inclusion criteria and were included in the quantitative synthesis (Figure 2). Across the 16 included studies, 1,666 patients were enrolled (range 22–224 per study).

The evidence base captured blood-based biomarkers spanning mechanistic strata along the immune–endothelial/BBB–glia–neuron axis (Gao and Hernandes, 2021; Liu et al., 2022), including upstream inflammation/innate immune candidates (e.g., TRAF6 and S100A8), intermediate BBB/glial markers (e.g., GFAP, S100β, including repeat measurements such as day-3 S100β), and downstream neuroaxonal injury markers (e.g., NSE, UCH-L1, Tau). Biomarkers were measured predominantly using immunoassays (ELISA or chemiluminescence), with study-specific positivity thresholds.

**Figure 3:**
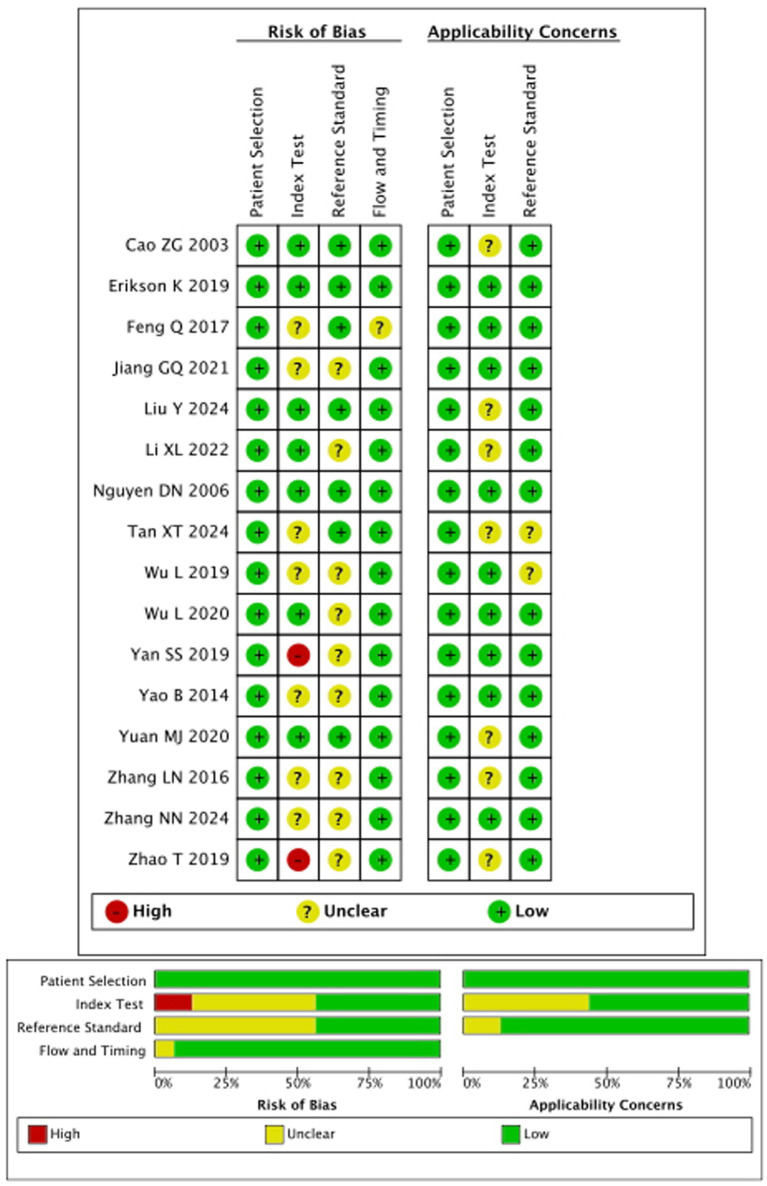
Risk of bias assessment using the QUADAS-2 tool for included diagnostic accuracy studies.

Importantly, heterogeneity was expected and prespecified, arising from two major sources: (i) SAE phenotyping criteria and (ii) sampling context. First, SAE reference standards varied across studies and were summarized using the prespecified tiering (Tier 1–3; Table 1). Second, sampling practices differed in both the anchor event (e.g., ICU admission, hospital admission, sepsis diagnosis, CAM-ICU assessment, or enrollment) and time-from-anchor (e.g., ≤24 h, 0–48 h, Day 1/Day 3 labels, or ≤72 h), which were extracted as an anchor-based sampling context rather than being forced into a single absolute “time window” (Table 1). Key study characteristics, biomarker classification, assay platforms, anchor events, time-from-anchor sampling context, and reconstructed diagnostic 2 × 2 contingency data are summarized in Table 1 and Supplementary Table S1. Risk-of-bias assessment using QUADAS-2 showed generally low-to-moderate concerns across the included studies. The most frequent source of unclear judgment was the index-test domain, where blinding of biomarker assessment or interpretation to SAE status was often not reported. Additional unclear judgments arose in the flow/timing domain because the interval between sampling and reference-standard assessment, sedation-interruption handling, and patient flow/exclusion reporting were incompletely described in several studies. Overall concerns regarding applicability were low (Figure 3) (Whiting et al., 2011).

### Diagnostic test accuracy meta-analysis

**Figure 4:**
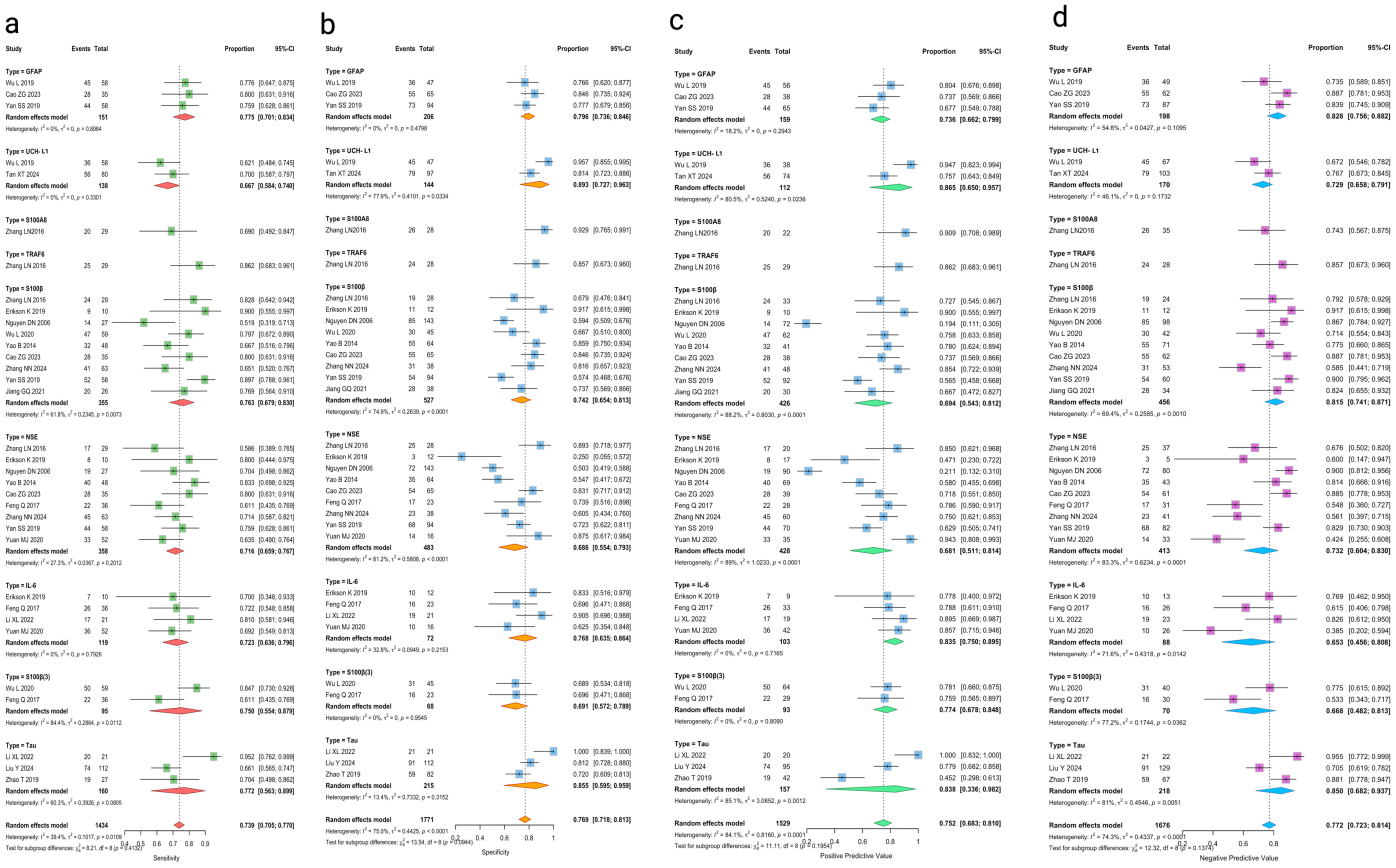
Forest plots of the pooled diagnostic accuracy estimates of biomarkers for sepsis-associated encephalopathy. **(a)** Sensitivity (95% CI); **(b)** specificity (95% CI); **(c)** positive predictive value - PPV (95% CI); **(d)** negative predictive value - NPV (95% CI).

**Figure 5:**
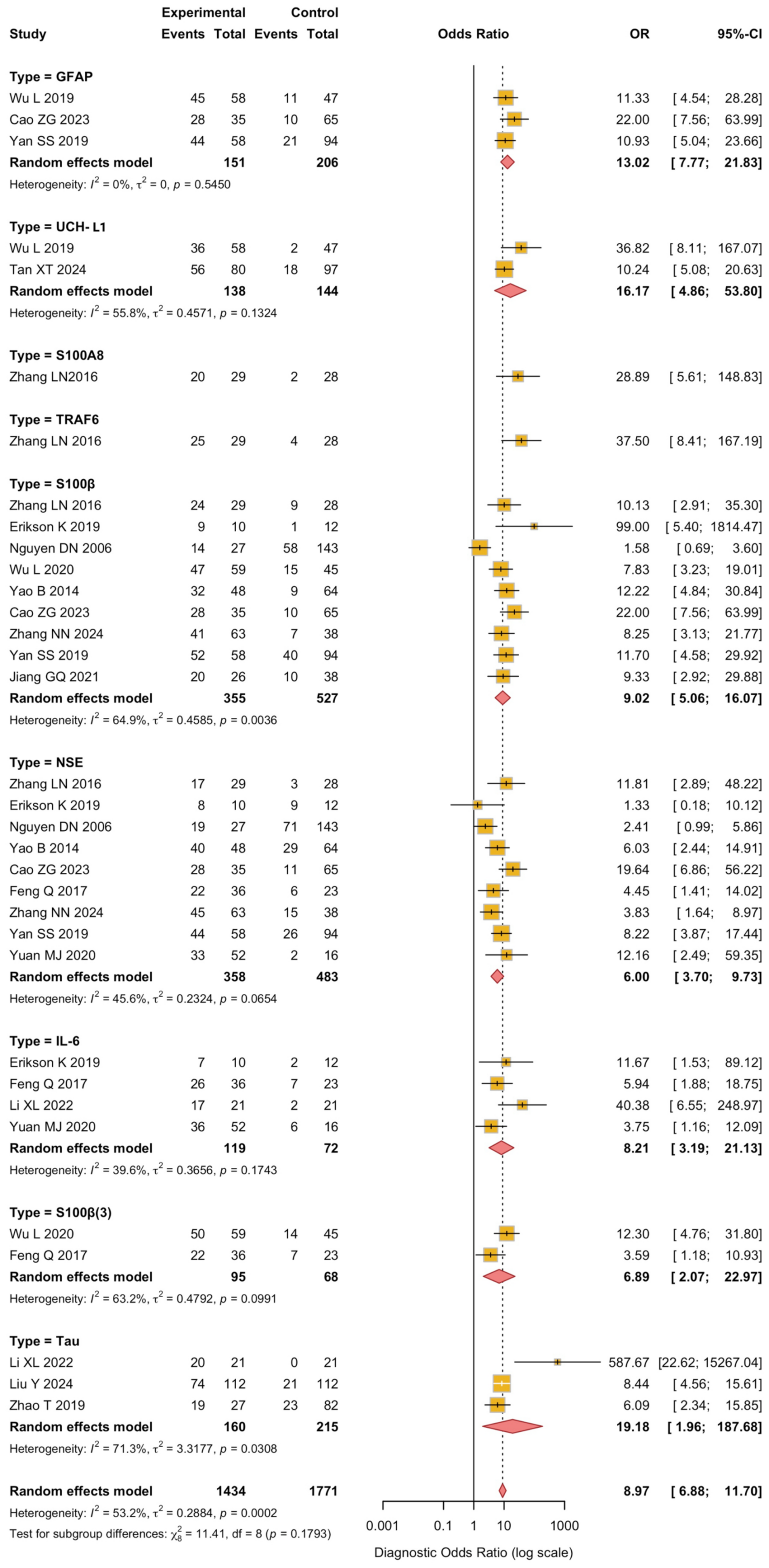
Forest plot of pooled diagnostic odds ratios (DORs) with 95% confidence intervals.

**Figure 6:**
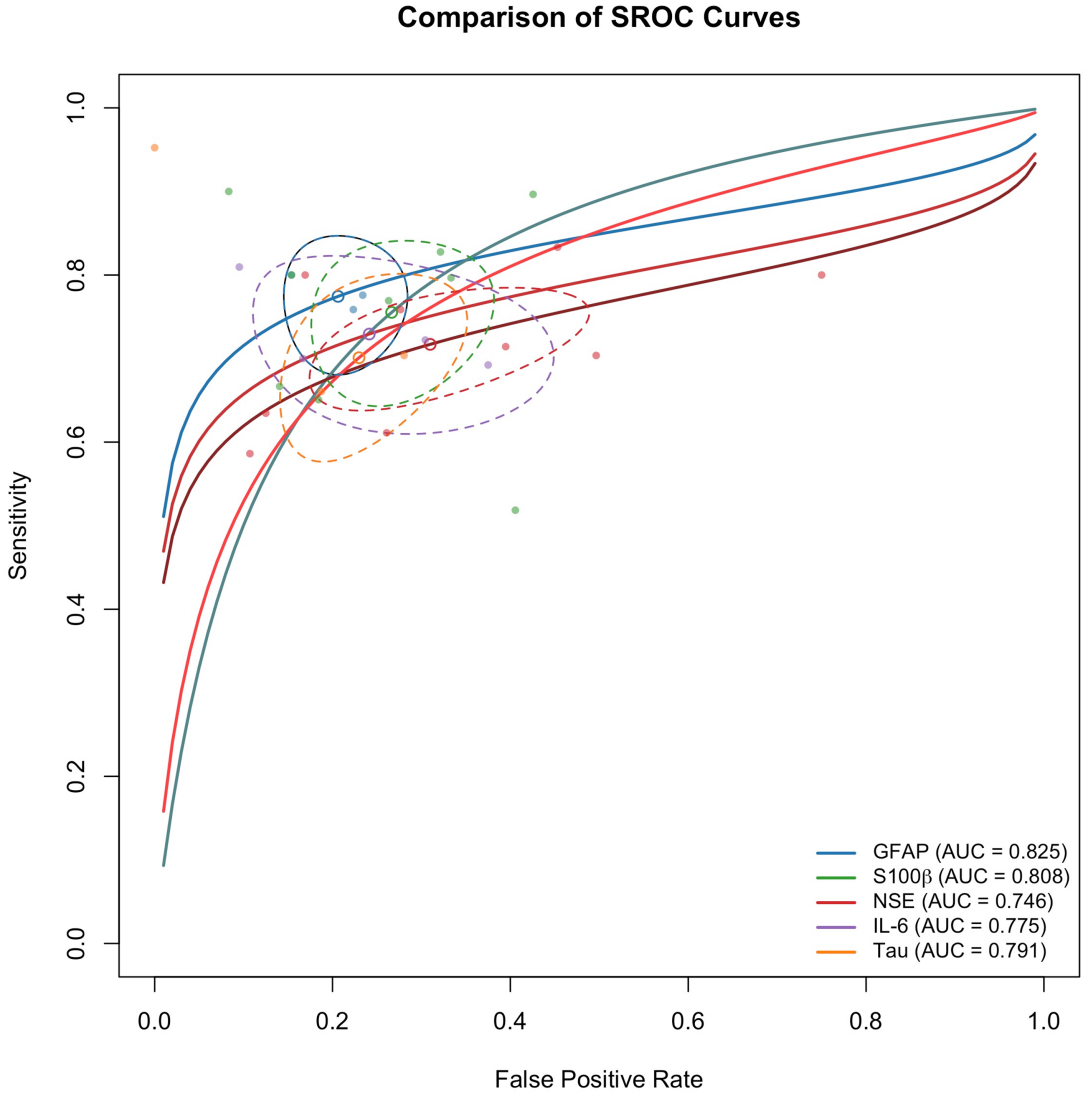
Summary receiver operating characteristic (SROC) curve for all included biomarkers.

Using a bivariate random-effects model, pooled diagnostic accuracy estimates (sensitivity, specificity, PPV, NPV, and DOR) were summarized for each biomarker. Forest plots for pooled sensitivity, specificity, PPV, and NPV are shown in Figure 4 (panels a–d), with pooled DORs presented in Figure 5. Overall discrimination is visualized by the SROC curve (Figure 6).

Pooled sensitivities ranged from 0.65 (UCH-L1; 95% CI 0.49–0.78) to 0.85 (TRAF6; 95% CI 0.65–0.96), and pooled specificities ranged from 0.67 (NSE; 95% CI 0.58–0.74) to 0.89 (S100A8; 95% CI 0.64–0.99). GFAP showed comparatively balanced performance, with a pooled sensitivity of 0.75 (95% CI 0.64–0.84) and specificity of 0.76 (95% CI 0.61–0.88) (Figure 4). PPVs ranged from 0.66 (UCH-L1) to 0.82 (S100A8), whereas NPVs ranged from 0.71 (NSE) to 0.86 (TRAF6). Because PPV and NPV are prevalence-dependent, pooled PPV/NPV should be interpreted cautiously given between-study variability in SAE prevalence. In terms of pooled discriminative ability, TRAF6 yielded the highest DOR (59.38, 95% CI 4.77–284.51), followed by S100A8 (DOR 47.66; 95% CI 2.97–238.50) and day-3 S100β (DOR 15.80; 95% CI 3.47–47.47) (Figure 5).

Because TRAF6 and S100A8 were supported by sparse clinical evidence (single-study nodes) and their thresholds/platforms were study-specific, these estimates should be interpreted as hypothesis-generating signals that prioritize upstream immune/alarmin candidates for prospective head-to-head validation rather than practice-ready conclusions. Across biomarkers supported by more than one study, glial-related markers (e.g., GFAP and S100β) tended to show higher and more balanced discrimination than classic neuronal injury indicators in the included settings, consistent with an immune–BBB/glia–neuron cascade interpretation.

SROC analysis yielded AUCs of 0.825 for GFAP, 0.808 for S100β, 0.791 for Tau, 0.775 for IL-6, and 0.746 for NSE, with an overall combined AUC of 0.86 (Figure 6). The overall AUC should be interpreted as a global summary of discrimination under hierarchical synthesis rather than a single-test estimate (Reitsma et al., 2005; Rutter and Gatsonis, 2001).

**Figure 7:**
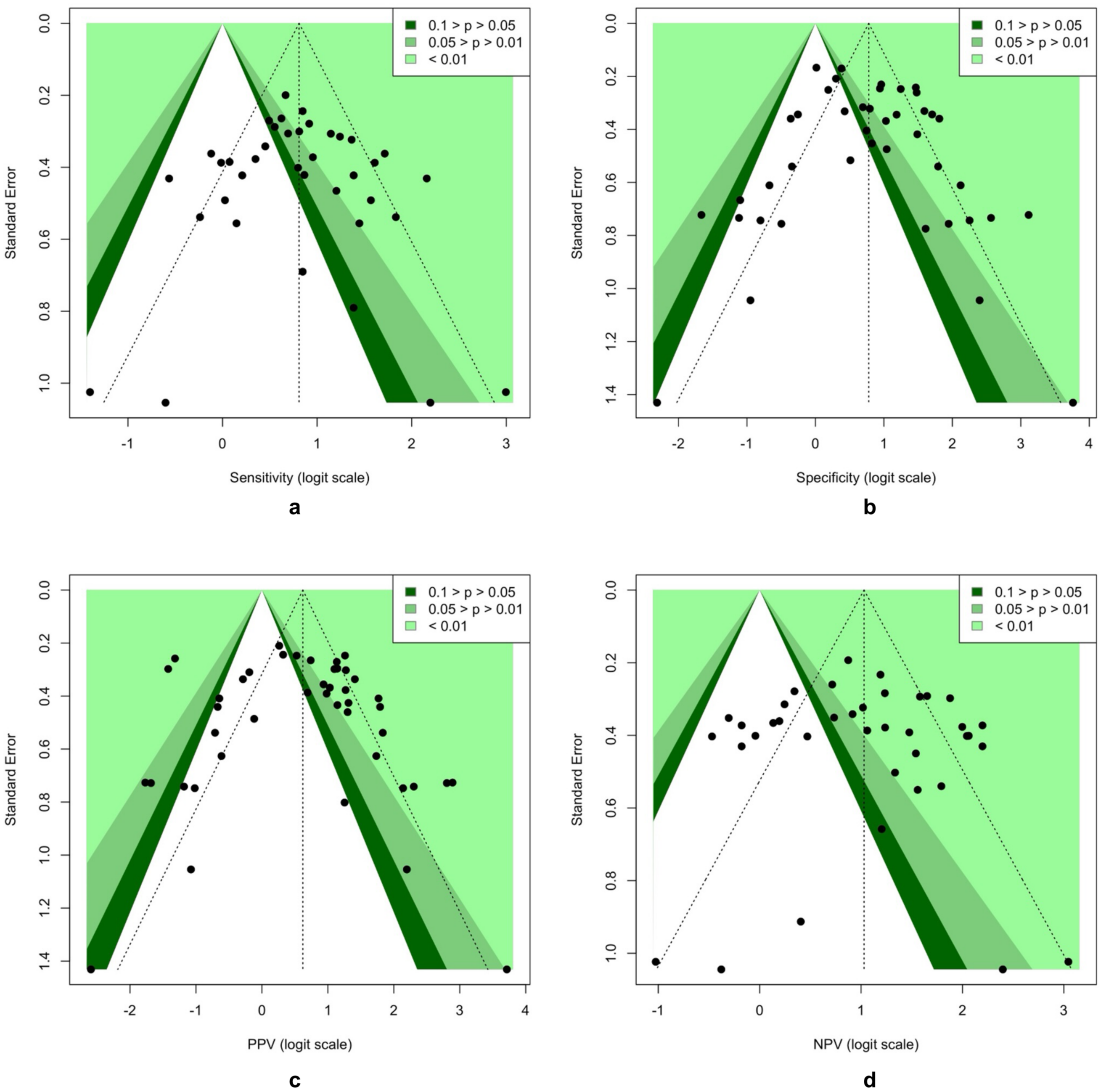
Funnel plots shown for descriptive visualization only: Sensitivity, specificity, PPV, and NPV. No formal statistical test for small-study effects (e.g., Deeks’ test) was performed because the number of studies per biomarker was limited; publication bias cannot be excluded.

Between-study heterogeneity was moderate to substantial (*I*^2^ 58–81%) (Higgins and Thompson, 2002), consistent with the prespecified sources of heterogeneity: reference-standard tiering (phenotyping differences), anchor-based sampling context (anchor event and time-from-anchor), as well as threshold and assay/platform variability (Table 1). Funnel plots are presented in Figure 7 for descriptive visualization only; no formal statistical test for small-study effects/publication bias (e.g., Deeks’ test) was performed due to limited study numbers per biomarker, and publication bias cannot be excluded (Duval and Tweedie, 2000).

### Bayesian diagnostic network meta-analysis

**Figure 8:**
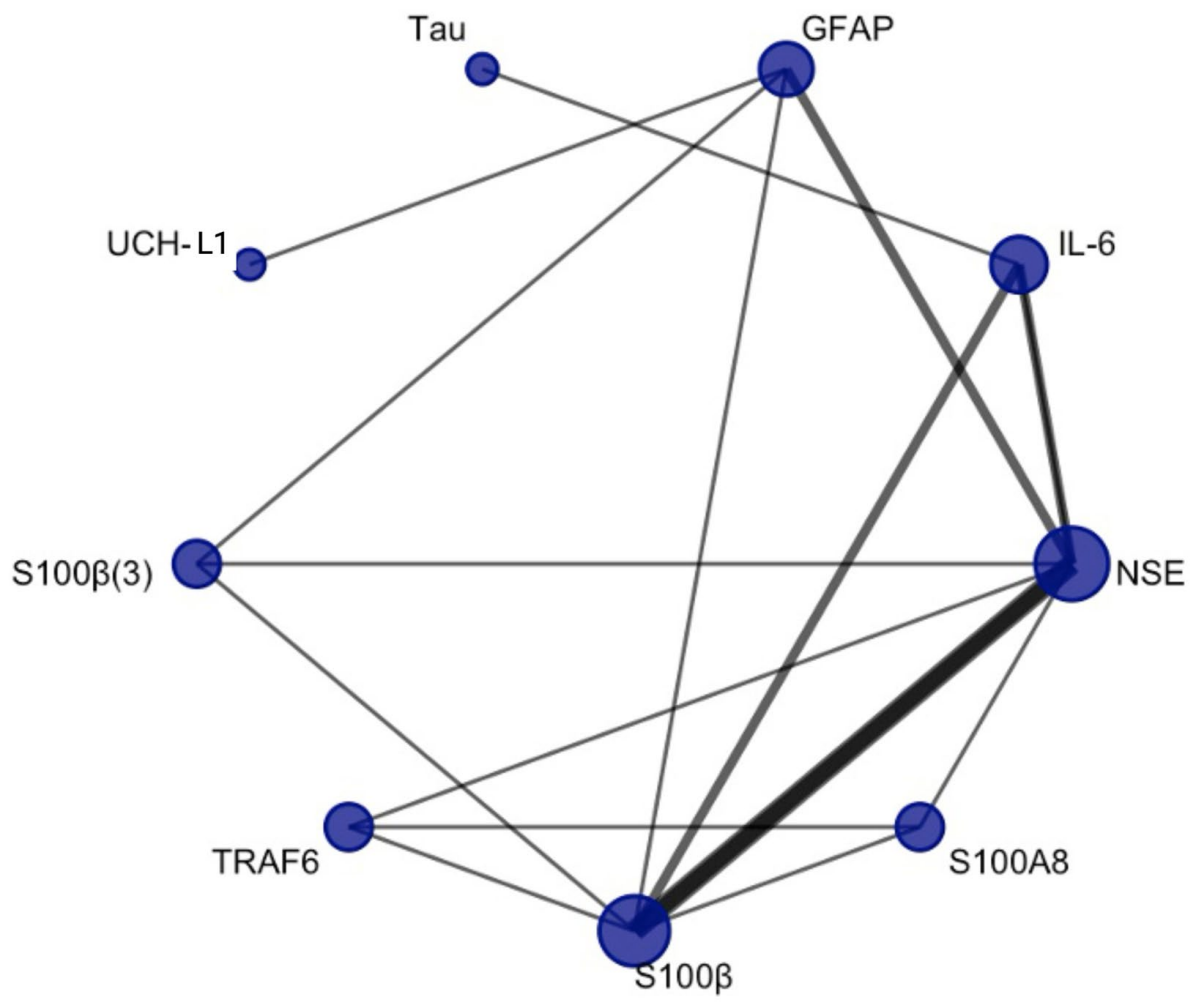
Network diagram displaying direct comparisons among biomarkers included in the Bayesian ANOVA model.

A Bayesian diagnostic network meta-analysis was conducted to integrate direct and indirect evidence across nine biomarker nodes. Network geometry is shown in Figure 8, illustrating available head-to-head comparisons and the connectivity that enables indirect comparisons under the hierarchical model. Consistent with our pathway and anchor-based sampling context framework, biomarker nodes represented study-level index tests (and timepoint-specific measurements when applicable, such as day-3 S100β), as detailed in Table 1 and Supplementary Table S1.

Based on the Advantage Index (S-value), TRAF6 ranked highest (S-value 8.84; 95% CI 0.14–17.00), followed by S100A8 (S 4.24; 95% CI 0.08–15.00) and day-3 S100β (S 3.84; 95% CI 0.09–13.00). NSE ranked lowest (S 0.20; 95% CI 0.07–1.00), with GFAP serving as the reference (S 2.42; 95% CI 0.09–11.00). Compared with GFAP, TRAF6 showed a relative sensitivity of 1.13 (95% CI 0.85–1.41) and a relative specificity of 1.06 (95% CI 0.67–1.42), whereas S100A8 showed a relative sensitivity of 0.90 (95% CI 0.59–1.22) and a relative specificity of 1.17 (95% CI 0.82–1.51) (Table 2). Given sparse connectivity and single-study support for selected nodes, comparative rankings should be interpreted as hypothesis-generating rather than definitive comparative performance.

**Table 2:** Comparative diagnostic accuracy and Bayesian ranking of blood-based biomarkers for sepsis-associated encephalopathy (SAE).

Marker	Sensitivity (95%CI)	Specificity (95%CI)	Diagnostic Odds Ratio (DOR)(95%CI)	Advantage index (S) (95%CI)	Relative sensitivity (95%CI)
GFAP	0.75 (0.64, 0.84)	0.76 (0.61, 0.88)	11.59 (4.02, 27.02)	2.42 (0.09, 11.00)	1
UCH-L1	0.65 (0.49, 0.78)	0.86 (0.70, 0.95)	16.21 (3.71, 44.55)	1.79 (0.09, 11.00)	0.87 (0.64, 1.10)
S100A8	0.68 (0.44, 0.86)	0.89 (0.64, 0.99)	47.66 (2.97, 238.50)	4.24 (0.08, 15.00)	0.90 (0.59, 1.22)
TRAF6	0.85 (0.65, 0.96)	0.80 (0.51, 0.96)	59.38 (4.77, 284.51)	8.84 (0.14, 17.00)	1.13 (0.85, 1.41)
S100β	0.73 (0.67, 0.79)	0.72 (0.64, 0.78)	7.14 (4.33, 11.14)	0.65 (0.08, 3.00)	0.98 (0.84, 1.17)
NSE	0.72 (0.65, 0.77)	0.67 (0.58, 0.74)	5.21 (3.12, 7.98)	0.20 (0.07, 1.00)	0.96 (0.82, 1.14)
IL-6	0.73 (0.62, 0.82)	0.74 (0.58, 0.87)	9.19 (3.10, 20.64)	1.38 (0.07, 9.00)	0.98 (0.79, 1.20)
Day-3 S100β	0.80 (0.68, 0.90)	0.74 (0.53, 0.89)	15.80 (3.47, 47.47)	3.84 (0.09, 13.00)	1.08 (0.88, 1.31)
Tau	0.74 (0.62, 0.85)	0.82 (0.67, 0.91)	15.82 (4.79, 40.20)	3.61 (0.09, 13.00)	0.99 (0.80, 1.24)

Comparative estimates should be interpreted as hypothesis-generating, particularly for biomarkers supported by single-study evidence.

## Discussion

Together, Figure 1, Table 1, and Supplementary Table S1 provide an integrated framework linking biomarker pathobiology, anchor-based sampling context (anchor event and time-from-anchor), and comparative diagnostic evidence synthesis. In this PRISMA-compliant systematic review and Bayesian diagnostic network meta-analysis (Page et al., 2021), we compared nine blood-based biomarkers for SAE across 16 studies and prioritized candidates using pooled discriminative metrics (including DOR) alongside Bayesian ranking approaches (Advantage Index, S-value). Three overarching observations emerged. First, biomarkers mapping to the immune–endothelial/BBB–glia–neuron cascade exhibited a coherent comparative hierarchy (Gao and Hernandes, 2021; Liu et al., 2022): upstream immune/alarmin candidates (TRAF6 and S100A8) yielded higher point estimates and rankings within a sparse evidence base (Wang et al., 2018; Zhang et al., 2016), while BBB/glial-related markers (GFAP and S100β) generally outperformed classic neuronal injury indicators (e.g., NSE) in the included diagnostic settings (Mazeraud et al., 2020; Wu et al., 2019; Zhao et al., 2019). Second, day-3 S100β consistently ranked among the leading candidates, a pattern that aligns with BBB/glial response kinetics rather than simply “later sampling” (Wu et al., 2020; Hu et al., 2023). Across the included literature, sampling was anchored to heterogeneous clinical events (e.g., ICU admission, sepsis diagnosis, or CAM-ICU assessment); when repeat measurements were available under the same anchor, the delayed measurement (typically labeled “Day 3”) may reflect a stage in which endothelial dysfunction and BBB disruption have amplified astroglial reactivity and neuroinflammatory signaling—processes that may be incompletely expressed at very early post-anchor sampling. Thus, the performance of day-3 S100β is best interpreted as anchor-context–dependent enrichment of BBB/glial biology rather than evidence that S100β is intrinsically a “late biomarker.” Third, neuronal/axonal injury markers (NSE, UCH-L1, Tau) likely function primarily as downstream severity signals: although many studies measured them within 72 h of the anchor event, these markers reflect neuroaxonal injury and may therefore be less informative for early case identification than for risk stratification in the included settings (Mazeraud et al., 2020; Gao and Hernandes, 2021; Liu et al., 2022; Wu et al., 2019; Zhao et al., 2019). Collectively, these findings support a pathway- and stage-informed view of SAE in which upstream innate immune/alarmin signals may complement conventional neuroglial and neuroaxonal injury markers, while the sampling anchor event and time-from-anchor materially shape which biological compartment is being “read out” by a circulating biomarker.

This distinction between sampling context and biological stage is critical for interpreting biomarker performance and for translating biomarkers into clinically meaningful diagnostic and risk-stratification strategies (Mazeraud et al., 2020). At the network level, comparative patterns were informed by the available evidence geometry (Figure 8) and relative diagnostic metrics versus the reference biomarker (Table 2). Importantly, comparative ranking in this synthesis should not be interpreted as immediate clinical superiority (Hu et al., 2023). TRAF6 and S100A8 were supported by single-study evidence within the current network, which can inflate apparent effect sizes, widen uncertainty, and may increase susceptibility to small-study effects; moreover, publication bias cannot be excluded. Mechanistically, alarmin-related signaling (e.g., S100A8–TLR4 pathways involving TRAF6) provides a biologically plausible upstream link to endothelial activation and BBB vulnerability (Gao and Hernandes, 2021; Schiopu and Cotoi, 2013). Accordingly, quantifying upstream immune mediators may provide an earlier window into SAE-related inflammatory cascades than downstream neuroglial or neuroaxonal injury markers in some settings (Liu et al., 2022). However, the high ranking of TRAF6 and S100A8 should be viewed as exploratory and hypothesis-generating, identifying candidates for priority investigation rather than biomarkers ready for routine clinical deployment. Our results therefore provide directional guidance for future research, not prescriptive clinical thresholds.

From a neurocritical care perspective, the clinical utility of any biomarker depends on whether it improves diagnosis or risk stratification beyond readily available predictors (e.g., delirium assessments, sedation exposure, and sepsis severity scores). Because most primary studies did not report multivariable models or incremental performance metrics, we could not quantify added value. Future prospective cohorts should predefine clinical predictor sets and assess incremental benefit using changes in discrimination and calibration and decision-analytic metrics (e.g., decision-curve analysis), ideally under standardized phenotyping and anchor-based sampling schedules.

Prior reviews in this area have often synthesized between-group biomarker concentration differences between SAE and non-SAE cohorts, which increases study coverage but does not directly support bedside diagnostic decision-making (Zenaide and Gusmao-Flores, 2013; Scarlatescu et al., 2025). By contrast, our analysis was explicitly framed as a diagnostic test accuracy synthesis, requiring extractable (or reconstructable) 2 × 2 data and focusing on clinically interpretable performance metrics (sensitivity, specificity, and DOR) alongside comparative Bayesian ranking (McInnes et al., 2018; Cohen et al., 2016). This design choice explains why fewer studies were eligible for quantitative synthesis and underscores a field-wide reporting limitation: many SAE biomarker studies do not provide prespecified thresholds, sufficient sampling descriptors (anchor events and time-from-anchor), or adequate information to reconstruct diagnostic contingency tables (Scarlatescu et al., 2025). Table 1 and Supplementary Table S1 therefore serve not only as summaries of included evidence but also as a transparent map of where incomplete reporting currently constrains cumulative inference and reproducible DTA synthesis.

Beyond comparative ranking, our findings outline a testable, pathway-informed research agenda for SAE. First, upstream immune/alarmin-related candidates (e.g., TRAF6 and S100A8) warrant prospective evaluation close to clinically relevant anchor events (e.g., ICU admission or sepsis diagnosis), with emphasis on early case detection and enrichment of screening cohorts (Zhang et al., 2016). Second, BBB/glial markers (e.g., GFAP and S100β) may benefit from standardized repeat-sampling designs anchored to the same clinical event, enabling characterization of endothelial–BBB disruption and delayed glial response dynamics that could optimize discrimination (Gao and Hernandes, 2021). Third, neuronal/axonal injury markers (e.g., NSE, UCH-L1, Tau) are more appropriately positioned as severity and prognostic enrichers rather than standalone tools for early SAE identification in the included settings (Wu et al., 2019; Gofton and Young, 2012). We propose that future prospective studies consider staged multi-marker panel designs aligned with biological compartments—immune activation, BBB/glial dysfunction, and neuroaxonal injury—using prespecified anchor events, harmonized sampling schedules, and predefined diagnostic thresholds (Cohen et al., 2016). Such designs would enable robust head-to-head comparisons within the same cohort, improve reproducibility of DTA synthesis, and accelerate translation toward clinically actionable SAE phenotyping strategies (McInnes et al., 2018).

This study has several limitations that should be interpreted as priorities for field-wide standardization and prospective validation.

First, heterogeneity in SAE reference standards remains unavoidable.

Phenotyping criteria varied across studies, and several reports relied primarily on delirium-focused tools, which may capture a narrower acute brain dysfunction phenotype rather than the full SAE spectrum (Ely et al., 2001; Bergeron et al., 2001; Gofton and Young, 2012). This variability can introduce differential misclassification and may contribute to between-study heterogeneity (Whiting et al., 2011). Because the available network was sparse across biomarker nodes, we did not perform formal tier-specific network comparisons, as such analyses might overstate precision. Instead, reference-standard tiering was used to make this heterogeneity explicit and to support cautious interpretation of the primary synthesis. Future studies should adopt harmonized SAE definitions with explicit exclusion of structural CNS disease, standardized sedation handling, and—where feasible—multimodal adjudication incorporating neurological examination and ancillary testing (e.g., EEG or neuroimaging) to improve phenotypic specificity (Hume et al., 2024).

Second, index-test thresholding and assay/platform differences limit cross-study comparability. Diagnostic cutoffs were often study-specific or incompletely prespecified, and assay platforms and specimen handling procedures were inconsistently reported, introducing potential threshold effects and measurement variability. Prospective studies should predefine thresholds (or provide complete ROC-derived threshold reporting), report assay kits/platforms and calibration procedures, and consider inter-laboratory harmonization or external quality control schemes to support reproducibility and clinically transferable decision thresholds (Cohen et al., 2016). In addition, pooled PPV/NPV estimates should be interpreted cautiously because they are prevalence-dependent and may not generalize across ICU case mix and SAE prevalence.

Third, sparse evidence for several biomarkers constrains the stability of comparative inference. Notably, TRAF6 and S100A8 were supported by single-study nodes, resulting in wide uncertainty and limiting confidence in comparative ranking despite high point estimates (Nyaga et al., 2018). Sparse connectivity can also increase susceptibility to small-study effects, and publication bias cannot be excluded. Adequately powered, prospective head-to-head studies that include upstream immune/alarmin candidates alongside established BBB/glial and neuronal injury markers within the same cohort are needed to strengthen comparative conclusions.

Fourth, residual clinical and contextual heterogeneity likely influenced biomarker distributions and performance. Differences in sepsis severity, ICU case mix, sedation practices, and sampling anchored to heterogeneous clinical events may contribute to variability that cannot be fully resolved in aggregate-data synthesis. We therefore emphasize an anchor-based sampling context framework and recommend prospectively standardizing anchor events (e.g., ICU admission vs. sepsis diagnosis) and time-from-anchor schedules to enable stratified analyses that separate biological-stage effects from sampling context.

Fifth, the incremental diagnostic or predictive value of biomarkers beyond established clinical predictors could not be assessed. Most primary studies did not report multivariable models that adjusted for key clinical covariates (e.g., sedation exposure, delirium screening results, and illness severity) or provide incremental performance metrics. Future studies should evaluate biomarker panels within prespecified clinical models and report both discrimination/calibration and decision-analytic measures to determine whether biomarkers add clinically meaningful power.

Collectively, these limitations reinforce that the present synthesis provides a mechanism-informed, hypothesis-generating prioritization of blood-based biomarkers for SAE. The most direct path toward translation is coordinated prospective validation with standardized phenotyping, prespecified thresholds, harmonized assays, and anchor-based sampling protocols. In addition, only English- and Chinese-language publications were included, which may have introduced language bias and may have led to omission of relevant studies published in other languages.

## Conclusion

This systematic review and Bayesian diagnostic network meta-analysis provides a mechanism-informed prioritization of blood biomarkers for sepsis-associated encephalopathy across an immune–BBB/glia–neuron axis. BBB/glial markers showed more balanced diagnostic discrimination, whereas upstream immune/alarmin candidates ranked highly but were supported by sparse data and study-specific thresholds. Prospective head-to-head studies are needed to test whether mechanism-staged biomarkers add incremental diagnostic value beyond established clinical predictors under standardized phenotyping and anchor-based sampling protocols.

## Data Availability

All data extracted or reconstructed are included in the article and its Supplementary material; additional data are available from the corresponding author upon reasonable request.

## Glossary

AUC: area under the curve
BBB: blood–brain barrier
CAM-ICU: Confusion Assessment Method for the Intensive Care Unit
CI: confidence interval
CrI: credible interval
DAMPs: damage-associated molecular patterns
DCA: decision curve analysis
DOR: diagnostic odds ratio
DTA: diagnostic test accuracy
EEG: electroencephalography
ELISA: enzyme-linked immunosorbent assay
FN: false negative
FP: false positive
FOUR: Full Outline of UnResponsiveness score
GFAP: glial fibrillary acidic protein
GCS: Glasgow Coma Scale
ICDSC: Intensive Care Delirium Screening Checklist
ICU: intensive care unit
I^2^: I-squared statistic (heterogeneity)
IL-1β: interleukin-1 beta
IL-6: interleukin-6
JAGS: Just Another Gibbs Sampler
MCMC: Markov chain Monte Carlo
NF-κB: nuclear factor kappa B
NPV: negative predictive value
NSE: neuron-specific enolase
PBMC: peripheral blood mononuclear cell
PPV: positive predictive value
PRISMA: Preferred Reporting Items for Systematic Reviews and Meta-Analyses
PRISMA-DTA: PRISMA extension for Diagnostic Test Accuracy reviews
R̂: Gelman–Rubin convergence diagnostic (potential scale reduction factor)
ROC: receiver operating characteristic
SAE: sepsis-associated encephalopathy
S100β (S100B): S100 calcium-binding protein B
S100A8: S100 calcium-binding protein A8
SOFA: Sequential Organ Failure Assessment
SROC: summary receiver operating characteristic
TLR4: Toll-like receptor 4
TN: true negative
TNF-α: tumor necrosis factor alpha
TP: true positive
TRAF6: TNF receptor–associated factor 6
UCH-L1: ubiquitin C-terminal hydrolase L1
