# Supplementary material for: Mechanism-informed diagnostic accuracy of blood biomarkers for sepsis-associated encephalopathy: a systematic review and Bayesian diagnostic network meta-analysis
Source: Front Mol Neurosci. 2026 Aug 3;19:1789013. doi: 10.3389/fnmol.2026.1789013 (PMC13479290; doi:10.3389/fnmol.2026.1789013)
Supplement: Supplementary file 1 [file Table_1.docx]

Supplementary Table S1. Reconstructed 2×2 diagnostic contingency data for blood-based biomarkers in sepsis-associated encephalopathy (SAE)

| First Author (Year) | Biomarker | TP | FP | FN | TN |
| --- | --- | --- | --- | --- | --- |
| Wu L,2019 | BBB/Glia:GFAP | 45 | 11 | 13 | 36 |
|  | Neuronal:UCH-L1 | 36 | 2 | 22 | 45 |
| Zhang LN,2016 | Immune:S100A8 | 20 | 2 | 9 | 26 |
|  | Immune:TRAF6 | 25 | 4 | 4 | 24 |
|  | BBB/Glia:S100β | 24 | 9 | 5 | 19 |
|  | Neuronal:NSE | 17 | 3 | 12 | 25 |
| Erikson K，2019 | BBB/Glia:S100β | 9 | 1 | 1 | 11 |
|  | Immune:IL-6 | 7 | 2 | 3 | 10 |
|  | Neurona:NSE | 8 | 9 | 2 | 3 |
| Nguyen DN，2006 | BBB/Glia:S100β | 14 | 58 | 13 | 85 |
|  | Neuronal:NSE | 19 | 71 | 8 | 72 |
| Wu L,2020 | BBB/Glia:S100β(Day 1) | 47 | 15 | 12 | 30 |
|  | BBB/Glia:S100β(Day 3) | 50 | 24 | 9 | 31 |
| Yao B,2014 | BBB/Glia:S100β | 32 | 9 | 16 | 55 |
|  | Neuronal:NSE | 40 | 29 | 8 | 35 |
| Cao ZG,2023 | BBB/Glia:GFAP | 28 | 10 | 7 | 55 |
|  | Neuronal:NSE | 28 | 11 | 7 | 54 |
|  | BBB/Glia:S100β | 28 | 10 | 7 | 55 |
| Feng Q,2017 | Neuronal:NSE | 22 | 6 | 14 | 17 |
|  | BBB/Glia:S100β  ( Day 3) | 22 | 7 | 14 | 16 |
|  | Immune:IL-6 | 26 | 7 | 10 | 16 |
| Zhang NN,2024 | Neurona:NSE | 45 | 15 | 18 | 23 |
|  | BBB/Glia:S100β | 41 | 7 | 22 | 31 |
| Yan SS,2019 | [BBB/Glia]GFAP | 44 | 21 | 14 | 73 |
|  | Neurona:NSE | 44 | 26 | 14 | 68 |
|  | BBB/Glia:S100β | 52 | 40 | 6 | 54 |
| Jiang GQ,2021 | BBB/Glia:S100β | 20 | 10 | 6 | 28 |
| Li XL,2022 | Immune:IL-6 | 17 | 2 | 4 | 19 |
|  | Neuronal:Tau | 20 | 0 | 1 | 21 |
| Liu Y,2022 | Neuronal:Tau | 74 | 21 | 38 | 91 |
| Tan XT,2024 | Neuronal:UCH-L1 | 56 | 18 | 24 | 79 |
| Yuan MJ,2020 | Neuronal:NSE | 33 | 2 | 19 | 14 |
|  | Immune:IL-6 | 36 | 6 | 16 | 10 |
| Zhao T,2019 | Neuronal:Tau | 19 | 23 | 8 | 59 |

**Notes:**Diagnostic contingency data (TP, FP, FN, TN) were extracted directly or reconstructed from reported sensitivity, specificity, and sample size when necessary.
Blood-based biomarkers include both circulating serum/plasma markers and PBMC-associated measurements.The anchor event denotes the clinical reference point for timing of blood sampling; “time from anchor” reflects study-reported intervals and does not imply uniform biological staging.Cutoff values and units are reported as in the original studies with harmonized formatting. Estimates from single-study biomarkers (e.g., TRAF6, S100A8) are interpreted as hypothesis-generating.
