# Supplementary material for: Mechanism-informed diagnostic accuracy of blood biomarkers for sepsis-associated encephalopathy: a systematic review and Bayesian diagnostic network meta-analysis
Source: Front Mol Neurosci. 2026 Aug 3;19:1789013. doi: 10.3389/fnmol.2026.1789013 (PMC13479290; doi:10.3389/fnmol.2026.1789013)
Supplement: Supplementary file 2 [file Table_2.docx]

Supplementary Table S2. Study-level clinical context, illness severity, sedation handling, and delirium assessment characteristics of uploaded primary studies

| **Study** | **Clinical setting / severity context** | **Sedation handling** | **Delirium assessment / frequency** | **Notes** |
| --- | --- | --- | --- | --- |
| **Wu L,2019** | Comprehensive ICU, Xiangya Hospital; sepsis cohort enrolled Jan 2015–Aug 2016; APACHE II and SOFA recorded within 24 h of ICU admission. | GCS evaluated before sedation; pre-sedation GCS used if sedated before ICU admission; daily spontaneous awakening trials; off-sedation evaluation window up to 24 h. | CAM-ICU plus RASS, twice daily until ICU discharge. | Sedation depth assessed daily; midazolam was more frequent in the SAE group. |
| **Zhang LN, 2016** | Central ICU cohort; baseline severity variables, including APACHE II and shock status, collected within 24 h of ICU admission. | Sedated patients underwent daily spontaneous awakening trials; if patients did not awaken within 24 h after sedation cessation, SAE was diagnosed. | CAM-ICU daily. | Strong study for ICU setting + sedation handling + delirium screening frequency. |
| **Erikson K, 2019** | Tertiary teaching hospital mixed medical-surgical ICU; septic shock only; APACHE II and SOFA metrics recorded. | Delirium assessment was performed only when RASS was −3 to +4. | CAM-ICU used; assessment frequency not explicitly reported. | Enter as CAM-ICU with RASS gating; frequency NR. |
| **Nguyen DN, 2006** | University hospital ICU cohort with severe sepsis/septic shock; severity assessed with APACHE III and daily SOFA. | Mechanically ventilated patients received continuous sedation (propofol or midazolam plus fentanyl, with neuromuscular blockade if required); GCS recorded before sedation. | No standardized delirium tool reported; encephalopathy confirmed clinically when symptoms persisted for 72 h after complete withdrawal of sedation. | Best described as clinical encephalopathy confirmation after sedation withdrawal. |
| **Wu L, 2020** | Comprehensive ICU, Xiangya Hospital; APACHE II and SOFA recorded within 24 h of ICU admission. | GCS evaluated before sedation; sedated patients managed with RASS plus daily spontaneous awakening trials; expected wake-up window within 24 h. | CAM-ICU twice daily from ICU admission to ICU discharge. | One of the clearest studies for reviewer-requested contextual variables. |
| **Yao B,2014** | Central ICU, Xiangya Hospital; sepsis cohort enrolled May 2012–Apr 2013; APACHE II and GCS collected within 24 h of ICU admission. | Daily spontaneous awakening trials in sedated patients; mental status reassessed within 24 h after sedation withdrawal. | Mental status assessed twice daily (09:00 and 18:00); no explicit CAM-ICU reporting. | Best described as clinical mental status assessment twice daily. |
| **Cao 2023** | Hospital-based sepsis cohort treated Aug 2021–Aug 2022; 65 sepsis and 35 SAE cases; exact ward type not explicitly stated in the accessible text; SAE severity/consciousness categories were stratified using GCS. | No sedation protocol reported in the accessible text. | No formal CAM-ICU or repeated delirium-screening schedule reported; SAE severity and consciousness state were classified using GCS. | Primary diagnostic study; appropriate to record ward type as NR if the full text does not specify ICU vs ward. |
| Feng Q,2017 | ICU cohort, Xiangya Hospital; APACHE II and SOFA compared between groups; biomarkers measured on ICU day 1 and day 3. | NR from currently accessible pages. | NR from currently accessible pages. | Use NR unless a fuller methods section becomes available. |
| Zhang NN,202 | Single-center prospective ICU cohort, Chifeng Municipal Hospital; APACHE II and SOFA recorded within the first 24 h of ICU admission. | For patients sedated before ICU admission, pre-sedation GCS was used; CAM-ICU performed only when RASS > −4. | Mental status assessed daily at 09:00 and 18:00; CAM-ICU plus RASS used for delirium assessment. | All patients also underwent daily TCD/ONSD assessment during hospitalization. |
| **Yan SS,2019** | ICU cohort, Third Xiangya Hospital; sepsis patients enrolled Oct 2016–Aug 2018; GCS, APACHE II and SOFA recorded within 24 h of ICU admission. | Sedative and muscle relaxant effects could not be fully excluded; CAM-ICU was used after sedative interruption. | CAM-ICU-based classification; scoring was performed only in the morning after stopping sedatives. | Retrospective study; authors explicitly noted residual sedative effect as a limitation. |
| Jiang GQ, **2021** | Hospital-based sepsis cohort from the Department of Emergency; patients enrolled Sep 2019–Sep 2020; SOFA and APACHE II recorded within 4 h of admission. | Patients were grouped in a non-sedated state. | No formal delirium instrument or repeated screening schedule reported. | Useful for severity context, but delirium-screening details are limited. |
| **Li XL,2022** | Retrospective burn sepsis cohort, Zhengzhou First People's Hospital; 41 burn patients with sepsis; burn area and APACHE II reported. | No explicit sedation protocol reported in the accessible text. | SAE defined by occurrence of consciousness/cognitive impairment during hospitalization after excluding direct CNS disease and metabolic/toxic causes; screening frequency NR. | Special population: burn sepsis, not a general ICU sepsis cohort. |
| **Liu Y,2024** | Hospital-based cohort, People's Hospital of Xinjiang Uygur Autonomous Region; 112 SAE vs 112 sepsis controls; severity stratified by GCS-based brain injury category and APACHE II risk category. | No sedation protocol reported in the accessible text. | Diagnosis based on attention/memory/orientation changes plus EEG and cranial CT; screening frequency NR. | Useful for severity-context fields rather than ICU delirium-screening workflow. |
| **Tan XT,2024** | Department of Geriatrics, Air Force 986th Hospital; elderly sepsis cohort; APACHE II and SOFA recorded within 24 h of admission. | Sedative/hypnotic causes of altered consciousness were excluded, but no formal sedation protocol was reported. | Diagnosis based on consciousness state plus cranial MRI and EEG, with exclusion of CNS infection, tumor, electrolyte disturbance, and sleeping-pill effects; screening frequency NR. | Special population: elderly sepsis cohort. |
| **Yuan MJ,2020** | ICU cohort, Foshan Second People's Hospital; sepsis patients enrolled Jan 2018–Jun 2019; APACHE II and SOFA collected. | Standard sepsis care included sedation and analgesia, but a specific sedation protocol was not reported. | SAE defined by acute mental status change (including delirium, consciousness disorder inconsistent with sedation, sleep-wake disturbance, hallucination, agitation); screening frequency NR. | Also included a treatment comparison of oral vs intravenous BCAA in SAE patients. |
| **ZhaoT, 2019** | Emergency ICU cohort, First Hospital of Jilin University; severe sepsis/septic shock; APACHE II, SOFA, GCS and infection sites collected within the first 24 h. | SAE required evaluation after complete withdrawal of sedation. | GCS and mental status were evaluated twice daily (08:00 and 18:00). | Strong study for severity context plus explicit assessment frequency. |

**Abbreviations:** ICU, intensive care unit; APACHE, Acute Physiology and Chronic Health Evaluation; SOFA, Sequential Organ Failure Assessment; GCS, Glasgow Coma Scale; CAM-ICU, Confusion Assessment Method for the ICU; RASS, Richmond Agitation-Sedation Scale; TCD, transcranial Doppler; ONSD, optic nerve sheath diameter; NR, not reported or not reliably extractable from the available full text/pages.

**Note:** The review article upload (file 7) was not included because it was not a primary diagnostic study. Duplicate uploads of the Feng 2017 article were used only to improve extraction accuracy. Cohorts were predominantly ICU-based, but several studies represented special populations (burn sepsis, elderly sepsis, or non-ICU hospital-based cohorts).
